# Dr. Mahlon R. Pritchard

**Published:** 1917-04

**Authors:** 


**Dr. Mahlon R. Pritchard,** College of Physicians and Surgeons of Baltimore, 1880, died at his home in Westfield, Pa., March, having become paralysed Feb. 2 and having a second attack Feb. 23. He was born in Westfield March 4, 1852. He studied medicine with Dr. Bottum of Westfield and took a part of his medical course at the Detroit College of Medicine. He practiced at Harrison Valley from 1880 till 1903, when he took a course at the Chicago Eye, Ear, Nose and Throat College, specialized along these lines, and located at Westfield.

